# Advanced Acoustic Monitoring Using Psychoacoustic Heatmap Machine Learning Models for Noise Impact Prediction in Air-Conditioned Building Environments

**DOI:** 10.3390/s26020544

**Published:** 2026-01-13

**Authors:** Kuen Wai Ma, Cheuk Ming Mak, Fu-Lai Chung, Hai Ming Wong

**Affiliations:** 1 Department of Mechanical Engineering, National Taipei University of Technology, Taipei 10608, Taiwan; 2 Department of Building Environment and Energy Engineering, The Hong Kong Polytechnic University, Hung Hom, Kowloon, Hong Kong, China; 3 Department of Computing, The Hong Kong Polytechnic University, Hung Hom, Kowloon, Hong Kong, China; 4 Faculty of Dentistry, The University of Hong Kong, Pok Fu Lam, Hong Kong Island, Hong Kong, China

**Keywords:** acoustic monitoring, machine learning, noise impact prediction, perceptual dimensions of sounds, psychoacoustic heatmap

## Abstract

Air-conditioning systems are vital for indoor environmental quality. However, noise can offset its benefits, making acoustic monitoring important. Recent research revealed that sound quality perceptions can be described by three psychological dimensions: *Evaluation*, *Potency*, and *Activity* (EPA). This is the first study to develop psychoacoustic heatmap machine learning models (PHMLM) for predicting sound quality and the negative noise impacts (*O1*: *Discomfortable*, *O2*: *Annoying*, *O3*: *Stressful*, and *O4*: *Unacceptable*) of air conditioning sounds using a 227 × 227-pixel psychoacoustic heatmap as input for machine learning. A total of 1208 jury listening tests were conducted with 101 participants on 30 s soundtracks from air-conditioned environments. Psychoacoustic heatmaps were generated by converting time-varying psychoacoustic metrics (*N*, *S*, *R*, and *FS*) into intensity maps containing 51,529 pixels of multidimensional acoustic information. The PHMLMs achieved predictive performance with correlation coefficients of 0.79, 0.80, and 0.62 for *E*-, *P*-, and *A*-scores, respectively. Compared to traditional regression models (TRM), PHMLM-EPA demonstrated significantly better performance with 31% lower mean absolute error (4.4 vs. 6.4) and higher regression slope (0.798 vs. 0.587). Moreover, PHMLM-EPA demonstrated a higher goodness-of-fit than TRM (+55% to +95%) and traditional acoustic metric *L*_Aeq_ (+87% to +95%). The approach offers an advanced acoustic monitoring method for sustainable building designs.

## 1. Introduction

Building acoustics is the science of noise control and prediction in buildings, studying the environmental sound quality effects and noise impacts on building occupants [1,2]. Owing to their function in improving indoor environmental quality, air-conditioning systems have become an essential component of modern buildings [3,4]. However, it is unavoidable that these systems produce noise from mechanical fans and/or the interaction of air flow turbulence with duct discontinuities such as dampers, sensors, bends, transition pieces, duct corners, and branch points [5,6,7]. Therefore, acoustic monitoring is essential to mitigate this issue [8,9,10]. The traditional assessment of air-conditioned noise environments relies on acoustic metrics such as Noise Criteria (*NC*), Noise Rating (*NR*), Room Criteria (*RC*), and A-weighted equivalent continuous sound pressure level (*L*_Aeq_) [11,12]. Although these metrics can provide single-value assessments of indoor noise environment acceptability, they fail to capture the noise characteristics and multidimensional psychological impacts on occupants beyond sensitivity to noise magnitude [13]. Traditional noise monitoring, prevention, prediction methods, and management strategies [14] have predominantly emphasized objective acoustic characterization, primarily due to the greater ease in predicting and measuring objective acoustic metrics compared to human subjective responses. Current insights highlight that the interactions between humans and their environments are essential for advanced acoustic monitoring in building acoustics. Therefore, multidimensional sound quality assessment [15] becomes essential for successful analysis of human–environment interactions and informed building design decisions.

Psychoacoustics is a branch of psychophysics to investigate the objective characteristics of indoor acoustic environments and the subjective perceptual influence on people. A psychoacoustic approach that takes both auditory and non-auditory effects of noise into account can provide a more accurate human perceptual judgement of noise. Psychoacoustic metrics are a series of the objective metrics to estimate the actual sensations of sounds based on the psychoacoustic scale (Bark scale) proposed by Eberhard Zwicker in 1961 [16]. Among these metrics, total loudness (*N*) stands out as the most recognized psychoacoustic metric, providing estimates of loudness sensation. The calculation methodology is standardized through ISO 532-1 [17]. The *N* measurement serves as a valuable complement to conventional acoustic measurement approaches in evaluating the energy characteristics of sounds. It was found that *N* had better performance than *NC* and *NR* in assessing the acoustic comfort of an indoor environment [18]. Furthermore, the spectral characteristics of sounds can be assessed through measurements of additional psychoacoustic metrics: sharpness (*S*), roughness (*R*), and fluctuation strength (*FS*) [19]. These metrics estimate sharpness sensation through calculation of energy skewness in sounds, roughness sensation by measuring rapid amplitude modulations, and fluctuation strength sensation by measuring slower amplitude modulations, respectively. The application of statistical sound levels such as *L*_A90_ and *L*_A10_, rather than time-equivalent sound levels, provides better identification of ambient noise levels vs. event noise levels by considering the temporal characteristics of sounds.

The interaction between humans and acoustic environments includes a series of processes extending from environmental sound generation and propagation through human perception of sound quality, ultimately resulting in various perceptual noise impacts. Despite the existence of ISO 15666 [20] and ISO 12913 [21,22,23] standards addressing the subjective evaluation of human perceptions, sound quality assessments demonstrate considerable variation across building acoustics studies [24,25]. The semantic differential scale [26], representing a widely applied psychological instrument for subjectively assessing the meaning of objective phenomena, provides quantitative evaluation of subjective perceptions. This scale involves constructing questions using bipolar semantic pairs representing contrasting meanings. A recent systematic review [27], examining applications of the semantic differential scale for subjective sound quality assessments [28,29], identified three fundamental human perceptual dimensions of sound: the *Evaluation*, *Potency*, and *Activity* (EPA) model. The *Evaluation* (*E*-), *Potency* (*P*-), and *Activity* (*A*-) dimensions correspond to general judgement, sensation of sound energy content, and sensation of temporal and spectral content of sounds, respectively. Therefore, a selection of acoustic metrics for environmental assessment should incorporate measurements relating to energy, spectral, and temporal content. The fundamental human perceptual dimensions of sound (*E*-, *P*-, and *A*-dimensions) were established through three components identified from a meta-analysis of a factor analysis of pooled data comprising 828,756 ratings on 1365 different sounds in the systematic review [27]. Research has also demonstrated that psychoacoustic metrics [30] and statistical noise levels [23] show significant consistency with perceptual assessment across the *E*-, *P*-, and *A*-dimensions.

In contrast to objective acoustic characterization, creating an effective, reliable, valid, and applicable psychometric tool for assessing multidimensional perceptual influence remains a continuing research focus. Various subjective assessment methodologies, along with their underlying factor models, have been developed across different studies [31]. However, analytical result discrepancies among these studies constrain the comparability of their findings. The Psychoacoustics Perception Scale (PPS) was consequently developed based on the EPA model to evaluate all necessary perceptions within the fundamental human perceptual dimensions of sound [15]. The identification of this sound quality model (EPA model) provides a valuable solution for addressing the challenge of extensive factor variation in multidimensional modelling of environmental sound quality.

In 2018, the World Health Organization (WHO) revised its environmental noise guidelines [32] to enhance public awareness regarding health impacts [33] of environmental noise exposure. Environmental noise is associated not only with discomfort but also with stress indicators, diseases, and mortality [34]. Additional noise impacts including discomfort [35], annoyance [36], and stress [37] from acoustic environments are widely recognized as having negative influences on individual mental health [38]. The EPA model provides a comparison framework between different studies and offers a foundation for predicting other negative noise impacts. Therefore, multidimensional sound quality assessments in this research included all acoustic and psychoacoustic metrics, PPS, and assessments of other negative noise impacts (*O1*: *Discomfortable*, *O2*: *Annoying*, *O3*: *Stressful*, and *O4*: *Unacceptable*) identified by WHO as associated with disease burden from environmental noise.

Artificial neural network (ANN) with feature learning is a type of machine learning algorithm to achieve artificial intelligence. ANNs were initially inspired by the biological neural networks that constitute human brains with numerous interconnected neurons [39]. The invention of applying non-saturating ReLU activation function into ANNs significantly increased the machine learning performance in image classification [40]. It accelerated the machine learning development and applications in data-driven research problems. After systematically searching for the keywords “Psychoacoustics” and “Machine Learning” in the ScienceDirect database, it was found that most of the 282 retrieved results focused on machine learning applications that use psychoacoustic metrics as input for objective signal classification tasks, such as the classification of heartbeats [41], vehicle sounds [42], conditions of heating system valves [43], vehicle fuel types [44], and sound type classification [45]. When the additional keyword “Noise Impact” was included (“Psychoacoustics” AND “Machine Learning” AND “Noise Impacts”), the number of search results dropped significantly to 12. After excluding three review papers and one study that mentioned the keywords only in the Section 4, five studies were identified that focused on objective classification tasks involving car engine sounds [46], tram gearbox conditions [47], bird species recognition [48], speech recognition [48], and railway pass-by detection [49]. Only three studies were found that applied machine learning using psychoacoustic metrics to predict subjective noise impacts, specifically in the contexts of aircraft noise [50], pedestrian noise [51], and noise within engineering machinery cabins [52]. These findings highlight a significant research gap in the development of smart, holistic, reliable, efficient, and user-friendly methods for assessing the multidimensional psychological impacts of air-conditioned noise environments on occupants. Traditional approaches fail to capture the temporal variations and spectral complexities that influence human perceptions of air-conditioned sounds. The EPA model’s incorporation of perceptual dimensions provides a more holistic assessment framework but requires innovative approaches to process the complex, time-varying acoustic data.

Among the three systematically reviewed studies that applied machine learning to predict subjective noise impacts, most focused on using time-averaged psychoacoustic metrics as input parameters for their models [50,51,52]. Although three of the five systematically reviewed studies that addressed objective classification tasks [46,47,48] employed classical acoustic spectrograms such as the Mel-spectrogram as model inputs, the comparative performance of acoustic and psychoacoustic spectrograms for noise impact prediction remains an unexplored research gap. This study represents the first investigation to integrate the EPA model concept and machine learning. This study addresses these challenges by introducing a novel psychoacoustic heatmap machine learning model (PHMLM) that transforms time-varying psychoacoustic metrics into comprehensive visual representations for noise impact prediction. The Mel-spectrogram is a classical visual representation of sound that displays energy distributions across the frequency–time domain for acoustic characterization. However, it does not account for human perceptual sensations such as loudness, sharpness, roughness, and fluctuation strength. Moreover, energy distributions in the Mel-spectrogram are derived from the fast Fourier transform and presented in constant frequency bands. It is well accepted that the human auditory sensation is in percentage frequency bands such as 1/3 octave bands or psychoacoustic scale (Bark bands). Preprocessing spectral-domain data into four psychoacoustic metrics (*N*, *S*, *R*, and *FS*) reduces the input data size by approximately 5000 times compared to that of the Mel-spectrogram. For instance, the Mel-spectrogram contains around 20,000 pixels for the 20 kHz audio frequency range, while the four psychoacoustic metrics require only four pixels. Thus, the multidimensional acoustic information related to energy, spectral, and temporal content in a 30 s soundtrack, influencing perceptual sensations, can be efficiently represented in a 227 × 227-pixel psychoacoustic heatmap with a temporal resolution of 0.002 s. These heatmaps serve as input to deep neural networks with transfer learning capabilities.

The principal objective of this research is to develop and validate PHMLM for automatically predicting sound quality and, hence, negative noise impacts in air-conditioned building environments from multidimensional objective acoustic characteristics. The significance of this research lies in providing a reliable, efficient, and user-friendly holistic psychoacoustic acoustic monitoring method that can inform sustainable building design and improve indoor acoustic environments by predicting occupant responses to air-conditioning noise using only 30 s recorded soundtracks.

## 2. Materials and Methods

### 2.1. Multidimensional Sound Quality Assessment

A holistic sound quality assessment [15,53] was found to be critical in assessing not only the objective energy, spectral, and temporal content of acoustic environments but also the subjective sound quality perceptions in *E*, *P*, and *A* dimensions of occupants. The multidimensional sound quality assessments were applied in this study to ensure that the psychoacoustic heatmaps covered all the multidimensional objective information of the acoustic environments and that the traditional regression model (TRM) achieved the largest reach in predicting subjective perceptual impacts.

#### 2.1.1. Multidimensional Objective Acoustic Characterization

**Figure 1:**
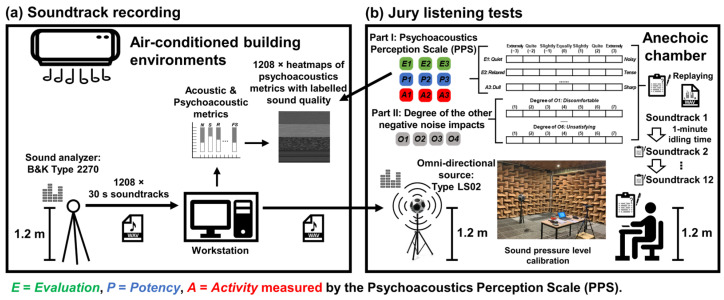
A flowchart of the experimental process: (**a**) preparing psychoacoustic heatmaps with labelled sound quality (**b**) from jury listening tests recording participants’ multidimensional subjective perceptual responses to replayed soundtracks in the study.

An advanced handheld analyzer (Type 2270; Bruel & Kjaer, Naerum, Denmark) was used to record sounds in air-conditioned building environments. A total of 1208 of 30 s soundtracks of different air-conditioned sounds were extracted from the sound recordings of more than 4000 min (see Figure 1a). Since *NC*, *NR*, and *RC* [11,12] are well accepted to be important in assessing acceptability of indoor environments, the acoustic characterization of the 30 s soundtracks extended the number of acoustic and psychoacoustic metrics from 21 to 24 in a multidimensional assessment proposed in the previous study [53] (see Table 1 and Table 2). Twelve (*L*_Zeq_, *L*_Aeq_, *L*_A10_, *L*_A50,_ *L*_A90_, *NC*, *NR*, *RC, L*_N_, *N*, *N*_5_, and *N*_95_), ten (*S*, *S*_5_, *S*_95,_ *R, R*_5_, *R*_95_, *FS*, *FS*_5_, *FS*_95_, and *f*_Mod_), and two (*L*_A10_–*L*_A90_ and *N*_5_–*N*_95_) metrics were applied to measure the energy, spectral, and temporal content of the soundtracks. The subscript number represents the metric’s percentile value. Acoustic metrics were directly obtained from the sound analyzer, while psychoacoustic metrics were calculated by using MATLAB, R2024a (MathWorks, Natick, MA, USA) software in free-field settings. The calculation of the psychoacoustics was based on the standard ISO 532-1 [17] and Zwicker’s book [16]. Equations of metric calculation and unit definition of the psychoacoustic metrics are listed in Appendix A.

#### 2.1.2. Multidimensional Subjective Perceptual Responses

All participants in the jury listening were university students at the Hong Kong Polytechnic University. Oral informed consent was obtained from all the participants, who were randomly selected on university campus prior to any assessment. No monetary compensation was provided for the listening tests. Only participants with normal hearing ability and living in typical daily environments were invited to the listening tests. Participants with self-reported chronic exposure to extremely noisy working or living environments or known hearing problems were excluded to eliminate potential uncontrollable confounds, as they have a higher likelihood of unnoticed hearing problems. Written informed consent was obtained, and demographic information (gender and age) was documented for each participant before the listening tests.

Jury listening tests were conducted in the anechoic chamber at the Hong Kong Polytechnic University (See Figure 1b) to collect multidimensional perceptual responses from participants regarding the replayed soundtracks. The anechoic chamber is an enclosed room with excellent sound insulation. The background noise level of the chamber was below 15 dBA with the low-frequency cut-off point of 80 Hz. In addition, the temperature of the studio can be controlled to maintain a temperature of 25 (±2 °C), and sufficient lighting is provided to control other indoor environmental factors than acoustics. The 30 s soundtracks were replayed during the listening tests using an amplified dodecahedron loudspeaker (Type LS02; Acoem, Limonest, France). For SPL calibration of the replayed soundtracks, a 1 kHz, 60 dB test tone at a 48 kHz sample rate was used. During the SPL calibration process, the 1 kHz test tone was played through an omni-directional source (Type LS02; Acoem, Limonest, France) at 1.2 m height, while the same handheld analyzer (Type 2270; Bruel & Kjaer, Naerum, Denmark) with a microphone was placed at ear height (1.2 m) of the listener’s position. The output level of the test tone was adjusted until the microphone reading was exactly 60 dB (reference for 60 phon true loudness level). The ‘calibrationFactor’ required for MATLAB-calculated psychoacoustic metrics can be obtained using the test tone signal and the 60 phon reference loudness level.

In the jury listening tests, participants were asked to complete a self-administered questionnaire based on their perceptual responses to each 30 s soundtrack. Each 30 s soundtrack was replayed repeatedly until participants completed the two parts of the questionnaire for each jury listening test. Each participant was required to complete eleven sets of a random 30 s soundtrack and a corresponding questionnaire, with a 1 min idling time between each set. All the soundtracks were randomly selected.

Psychoacoustics Perception Scale (PPS) comprised Part I of the self-administered questionnaire to quantify participants’ perceptual responses in *E-*, *P-*, and *A*-dimensions of sound. There were nine questions (*E1*: *Quiet-Noisy*, *E2*: *Relaxed-Tense*, *E3*: *Pleasant-Unpleasant, P1*: *Quiet-Loud*, *P2*: *Light-Heavy*, *P3*: *Weak-Strong, A1*: *Deep-Metallic*, *A2*: *Low-High*, and *A3*: *Dull-Sharp*) in a 7-level semantic differential scale [3,15]. For example, question *P1: Quiet-Loud* is constructed using a pair of opposite perceptions, ‘Quiet’ and ‘Loud’, on a 7-level scale ranging from ‘Extremely Quiet (−3)’, ‘Quite Quiet (−2)’, ‘Slightly Quiet (−1)’, ‘Equally (0)’, ‘Slightly Loud (1)’, ‘Quite Loud (2)’, and ‘Extremely Loud (3)’. Cronbach’s α reliability tests [54] were conducted to check the internal consistencies of the sematic pairs in the *E-*, *P-*, *A-*, and *EPA*-dimensions for the approach of summing the item scores of the semantic pairs into a factor score of a dimension. The degree of the internal consistency was represented by the reliability coefficient (Cronbach’s α). Cronbach’s α > 0.90, 0.80, 0.70, or 0.60 shows an excellent, good, acceptable, or questionable internal consistency of the questions in the *E-*, *P-*, and *A-*dimensions [54]. The *E-*, *P*-, *A*-, and *EPA*-scores were the summation of the items in *E-*, *P*-, *A*-, and *EPA*-dimensions, respectively.

Four questions of assessing the degree of the other negative noise impacts (*O1*: *Discomfortable*, *O2*: *Annoying*, *O3*: *Stressful*, and *O4*: *Unacceptable*) in a 7-level Likert scale comprised Part II of the self-administered questionnaire. Higher scores on questions *O1*–*O4* indicated greater discomfort, annoyance, stress, and lack of acceptance with the replayed sounds.

### 2.2. Psychoacoustic Heatmap Machine Learning Model (PHMLM)

#### 2.2.1. Psychoacoustic Heatmaps of 30-s Soundtracks

**Figure 2:**
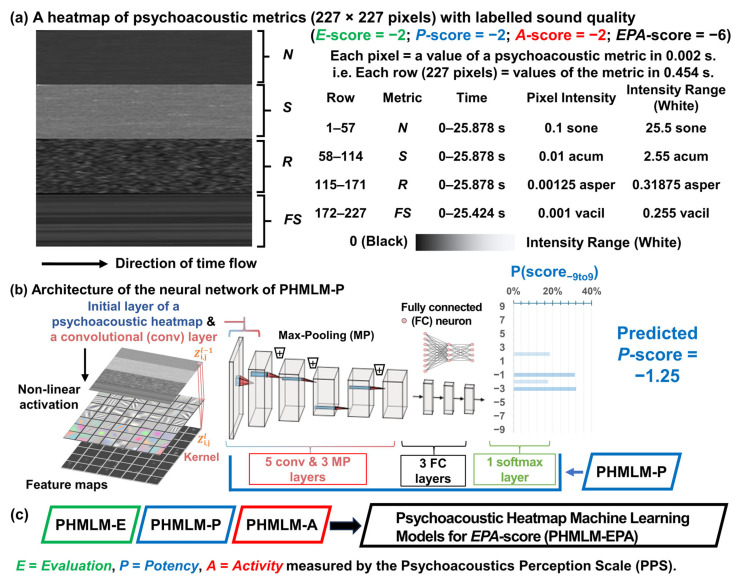
A schematic diagram of the development of psychoacoustic heatmap machine learning models (PHMLM): (**a**) an example of a psychoacoustic heatmap; (**b**) architecture of the neural network of PHMLM-E; and (**c**) a structure of PHMLM-EPA.

A psychoacoustic heatmap with labelled sound quality in terms of *E-*, *P*-, and *A*-scores was prepared for each 30 s soundtrack containing air-conditioning sounds. Each psychoacoustic heatmap was prepare in 227 × 227 pixels in grey scale (see Figure 2a). The size of 227 × 227 pixels was selected to match the standard input resolution of the AlexNet architecture [40] used in transfer learning in this study (see Section 2.2.2). This choice allowed direct use of pre-trained convolutional layers without resizing operations that could distort the underlying patterns of psychoacoustic heatmaps. Each pixel in the psychoacoustic heatmaps represented a psychoacoustic metric value in 0.002 s which is the time resolution for calculating time-varying loudness as stated in ISO 532-1 [22]. Since each soundtrack was recorded in 48 kHz sample rate of 24 bits per sample, every 96 samples were used to calculate a psychoacoustic metric in 0.002 s. Each row of psychoacoustic heatmaps represented the values of a psychoacoustic metric in 0.454 s (227 × 0.002 s). The four most commonly applied psychoacoustic metrics (*N*, *S*, *R*, and *FS*) in environmental noise studies were selected according to a systematic review [55]. The four psychoacoustic metrics in a psychoacoustic heatmap were ordered by their adoption rate of the metrics (*N*: 23%, *S*: 21.9%, *R*: 16.3%, and *FS*: 14%) in environmental noise studies. The first 57 rows were the values of the psychoacoustic metric *N* in 25.878 s (57 × 0.454 s). The second and third 57 rows, respectively, were the values of the psychoacoustic metrics *S* and *R* in 25.878 s. The last 56 rows were the values of the psychoacoustic metric *FS* in 25.424 s (56 × 0.454 s). The psychoacoustic heatmaps were normalized globally across the entire dataset using fixed maximum intensities. The maximum intensities of the psychoacoustic metrics (*N*, *S*, *R*, and *FS*) were determined by the upper bound of the 95% CI of the 5th-percentile values, with a safety factor of at least 2 to prevent data loss in extreme cases. For example, the upper bound of the 95% CI of the 5th-percentile value for metric *N* (*N*_5_) was 9.12 sone (see Table 2); thus, the maximum intensity was set to 25.5 sone (25.5 > 18.24 = 9.12 × 2). Similarly, the maximum values for metrics *S*, *R*, and *FS* were set to 2.55 acum (>2.5 = 1.25 × 2), 0.31875 asper (>0.26 = 0.13 × 2), and 0.255 vacil (>0.08 = 0.04 × 2), respectively. Since pixel intensities span 0 to 255 (256 grayscale levels, where 0 represents black and 255 represents white), this corresponds to per-pixel intensities of 0.1 sone, 0.01 acum, 0.00125 asper, and 0.001 vacil, respectively.

#### 2.2.2. Architecture of Neural Network for Machine Learning

The psychoacoustic heatmap machine learning model (PHMLM) was designed based on the architecture of the neural network AlexNet [40] that achieved high performance in image classification tasks by introducing a non-saturating ReLU activation function. An architecture of all PHMLM was constituted by the five convolutional (conv) layers, three max pooling (MP) layers, three fully connected (FC) layers, and one softmax layer (see Figure 2b). In each conv layer, noise features were extracted through sequential convolution kernels and non-linear activation layers.

(1) $$Z_{i,j}^{l}=\max\left({0,\;W}^{T}Z_{i,j}^{l-1}+b\right),$$

where $Z_{i,j}^{l}$ $Z_{i,j}^{l-1}$, ***W***, ***b***, *y*+ = *max*(0,*y*) were the convolution features at a location (i,j) at layer *l*, input centred at (i,j) from the previous layer *l* − 1, kernel weights, kernel bias, and ReLu activation function, respectively [56]. The output of a conv layer is a feature map to store the extracted features at locations. In each MP layer, a feature map was downsized by extracting the most informative features at small regions.

(2) $$Z_{k}^{l}=\underset{Z\in s}{\max}Z_{k}^{l-1},$$

where $Z_{k}^{l}$ is the k-th output of the MP layer *l*, $Z_{k}^{l-1}$ is the feature output from the previous layer *l* − 1, and *s* is the pooling size. The noise features of the last MP layer were flattened into a vector as the input of the first FC layer. In each FC layers, high-level features were stored in the artificial neurons that fully connect to all neurons in the previous *FC* layer. Sigmoid activation function *S*(*x*) was used in the *FC* layers.

(3) $$S\left(x\right)=\frac{e^{x}}{e^{x}+1},$$

(4) $$Z^{l}=S\;(\sum_{u.v\;}{{(W}^{l})}^{T}Z^{l-1}+b^{l}),$$

where $W^{l}\;\in R^{U\times V}$ is the weighted matrix of the FC layer, u = 1,2, …, U, v = 1, 2, 3, …, V, $Z^{l-1}$ is the input feature of the FC layer *l*, and ***b****^l^* is the bias vector. The pre-trained architecture [40] was loaded by using MATLAB, R2024a (MathWorks, Natick, MA, USA). The transfer learning approach involved extracting all layers except the final three classification layers (layers 1 to end-3). These transferred layers served as feature extractors for the psychoacoustic heatmaps. The architecture of PHMLM-E, PHMLM-P, and PHMLM-A was then modified by adding new classification layers specifically designed for the 19-level *E-*, *P*-, and *A*-scores, respectively. A new FC layer was added with the number of outputs equal to 19 levels (−9 to 9), followed by a softmax layer for probability distribution and an output layer for final classification (labelled *E-*, *P*-, or *A*-scores from the jury listening tests). The newly added FC layer was configured with enhanced learning rates (WeightLearnRateFactor = 20, BiasLearnRateFactor = 20) to accelerate the learning of task-specific features while maintaining the pre-trained convolutional features.

#### 2.2.3. Machine Learning Training

The 5-fold cross-validation method [57] was applied to prevent the overfitting of the model. The entire dataset was divided into five stratified folds using MATLAB’s ‘splitEachLabel’ function with the option ‘randomized’. This created five datasets where each fold randomly samples 20% of psychoacoustic heatmaps from every label, enabling 5-fold training while preserving class distributions. The training process was run five times with the 1st Training (T1) using the 1st 20% of the psychoacoustic heatmaps with labelled sound quality as the 1st validation set (and the remained 80% as the training set), the 2nd Training (T2) using the 2nd 20% of the psychoacoustic heatmaps as the 2nd validation set, and so on. Training was configured using Stochastic Gradient Descent with Momentum (SGDM) optimization. All hyperparameters in the ‘trainingOptions’ using MATLAB R2024a (MathWorks, Natick, MA, USA) were carefully selected based on empirical testing and included the following (see Table 3): mini-batch size of 80 psychoacoustic heatmaps per iteration to balance computational efficiency and gradient stability, maximum of 4000 epochs to ensure sufficient training iterations, initial learning rate of 1 × 10^−3^ for optimal convergence, validation frequency of every 12 iterations for continuous performance monitoring, and momentum coefficient of 0.95 to accelerate convergence and reduce oscillations. A piecewise learning rate schedule was implemented with learning rate reduction every 400 epochs by a factor of 0.6 to facilitate fine-tuning in later training stages. The piecewise learning rate schedule requires 4000 epochs for 10 decay steps (drop every 400 epochs × 0.6 factor), ensuring fine convergence of psychoacoustic heatmap features without premature stagnation. The training dataset was shuffled at every epoch to prevent overfitting to data order patterns. In every training iteration, a prediction error was be returned by the loss function.

(5) $${Loss}_{batch}=-\frac{1}{n}\times \sum_{i}^{n}\sum_{j=1}^{19(-9to9)}{score}_{i,j}\times log(P\left({score}_{i,j}\right)),$$

where *n* is the number of samples in each batch, score_i,j_ is 1 if sample i belongs to Score_j_, and P(score_−9 to 9_) is the predicted probability of *E-*, *P*-, or *A*-scores (−9 to 9). The network weights of the layers were updated per batch to obtain a smaller prediction error. Twelve mini batches were included in each epoch, a completed loop of the whole training dataset.

(6) $$Loss=\sum_{b=1}^{batch}{Loss}_{b}/batch.$$

The final output layer of each PHMLM-E, PHMLM-P, and PHMLM-A is the softmax layer to prove the probability, P(score_−9 to 9_), of the assigned −9 to 9 *E*-, *P*-, and *A*-scores, respectively.

(7) $$Predicted\;score=\sum_{i=1}^{19(-9to9)}\left(i\times P\left({score}_{i}\right)\right),$$

The final PHMLM-EPA was the overall sound quality prediction that summarizes all the predicted scores from PHMLM-E, PHMLM-P, and PHMLM-A (see Figure 2c). Mean absolute error (MAE) was calculated to measure the errors between the predicted and labelled scores of sound quality.

(8) $$MAE=\sum_{i=1}^{n_{PH}}\left(\left|Predicted\;score_{i}-Labelled\;score_{i}\right|/n_{PH}\right),$$

where n_PH_ is the number of the psychoacoustic heatmaps.

### 2.3. Statistical Analysis

All the measured objective acoustic characteristics and subjective perceptual responses were coded. All two-tailed statistical tests in this study were conducted using SPSS, version 26.0 (IBM Corp., Armonk, NY, USA) at a significance level of 0.05 [58]. The normality of all data was assessed by Shapiro–Wilk tests [59]. Spearman’s rank correlation tests (non-parametric, rank-based bivariate correlation tests) were conducted to determine Spearman’s rank correlation coefficient (*ρ*, Equation (A10)) between variables when objective data were not normally distributed. Since the model-predicted subjective scores cannot be assumed to maintain the same distributional properties as the labelled subjective scores, non-parametric Spearman’s rank correlation tests were chosen to evaluate model predictive performance, even though the labelled subjective scores followed normal distributions.

For PHMLM-EPA model validation, the traditional regression model for *EPA*-score prediction (TRM-EPA, Equation (A11)) served as the validation baseline. The TRM-EPA was constructed using stepwise linear regression, a standardized analysis method [23] recommended by ISO 12913-3 [23] for environmental noise studies involving subjective data. This method was more appropriate as a validation baseline than other machine learning methods, which show heterogeneity across studies. In a stepwise regression, potential significant predictors are added or removed based on a significance level of 0.05. The coefficient of determination (adjusted *R*^2^, Equation (A12)) was an indicator of the goodness-of-fit of a model.

## 3. Results

### 3.1. Descriptive Statistics of Sound Quality Assessments

A total of 120 university students were randomly selected on campus. Fourteen reported chronic exposure to extreme noisy environments (markets (*n* = 3), road traffic (*n* = 5), and construction work (*n* = 6)), and five had known hearing impairments (tinnitus (*n* = 2) and middle-ear impairment (*n* = 3)). Therefore, 101 participants were included in the listening tests. Of these, 72 (71.3%) were male and 29 (28.7%) were female, with a mean age of 22.6 years (SD = 2.1). After excluding four incomplete listening tests, 1208 valid jury listening tests were completed (101 participants × 12 tests − 4 tests).

#### 3.1.1. Multidimensional Objective Acoustic Characteristics

Table 2 and Table 3 presented descriptive statistics for 24 acoustic and psychoacoustic parameters measured from 1208 soundtracks recorded in air-conditioned building environments such as lecture halls, university classrooms, offices, and libraries. None of the metrics followed a normal distribution, as indicated by the Shapiro–Wilk tests (all *p*-values < 0.05). The median values of the conventional acoustic metrics (*L*_zeq_, *L*_Aeq_, *L*_A10_, *L*_A50_, *L*_A90_, *NC*, *NR*, and *RC*) closely matched their means, suggesting a generally symmetrical distribution despite the lack of normality. The interquartile range (IQR) of the conventional acoustic metrics was about 9 dBA. The small mean values were found for *L*_A10_*–L*_A90_ (0.94 dBA) and *N*_5_*–N*_95_ (0.99 sone).

For the psychoacoustic metrics *N*, *S*, *R*, and *FS*, the 75% percentile values of *N*_5_, *S*_5_, *R*_5_, and *FS*_5_ were 10.44 sone, 1.35 acum, 0.15 asper, and 0.05 vacil, respectively. Both of them were found to be much smaller than the maximum values (25.5 sone, 2.55 acum, 0.31875 asper, and 0.255 vacil) of the metrics in psychoacoustic heatmaps. The dominant modulation frequency (*f*_Mod_) was found to be 131 (±standard deviation = 31 Hz).

#### 3.1.2. Multidimensional Subjective Perceptual Responses to the Replayed Sounds

**Figure 3:**
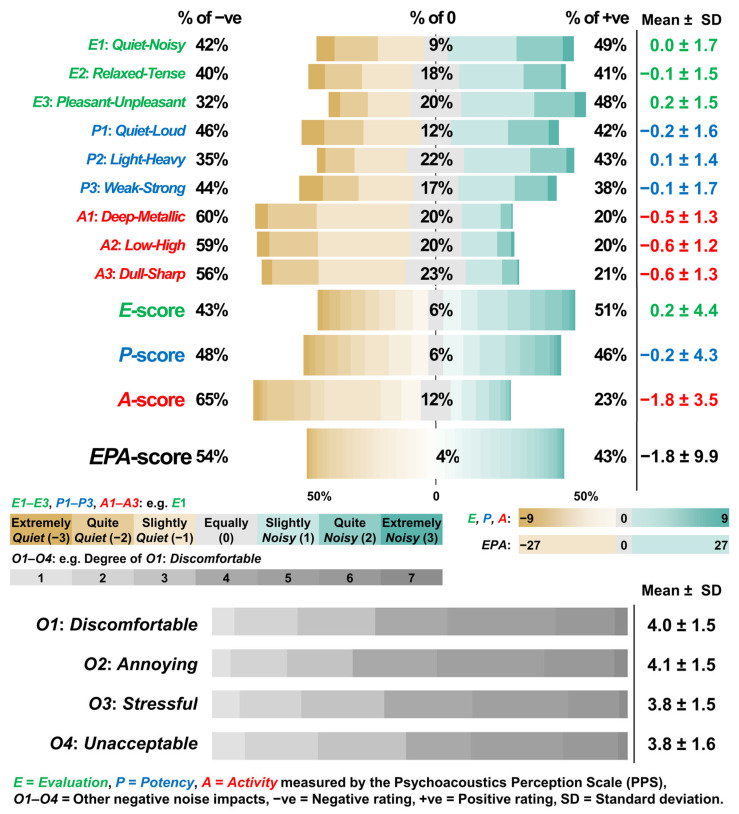
Horizontal stacked bar charts of the perceptual data of the soundtracks (*n* = 1208) captured using Psychoacoustics Perception Scale (PPS) in *Evaluation*, *Potency*, and *Activity* (*EPA*) dimensions and four questions about other negative noise impacts (*O1*–*O4*).

In general, the means of the questions in *E-* and *P-*dimensions were close to zero, while the means of the questions in *A* dimension was in negative values (see Figure 3). Therefore, the means of the *E*-, *P*, and *A*-scores were 0.16 (± 4.4), −0.22 (± 4.3), and −1.77 (± 3.5), respectively. The mean of the *EPA*-score was also found to be negative at −1.83 (± 9.9). In the responses of *O1*–*O4*, the degree of *O1*: *Discomfortable* (4.0 ± 1.5) and *O2*: *Annoying* (4.1 ± 1.5) was found to be higher than that of *O3*: *Stressful* (3.8 ± 1.5) and *O4*: *Unacceptable* (3.8 ± 1.6). The normality tests (Shapiro–Wilk tests) results for the *E*-, *P*-, *A*-, and *EPA*-scores were found to be non-significant (*p*-values ≥ 0.05). All the labelled subjective scores were normally distributed despite their negative means.

### 3.2. Psychoacoustic Heatmap Machine Learning Model (PHMLM)

#### 3.2.1. Predictive Performance of PHMLM-E, PHMLM-P, and PHMLM-A

**Figure 4:**
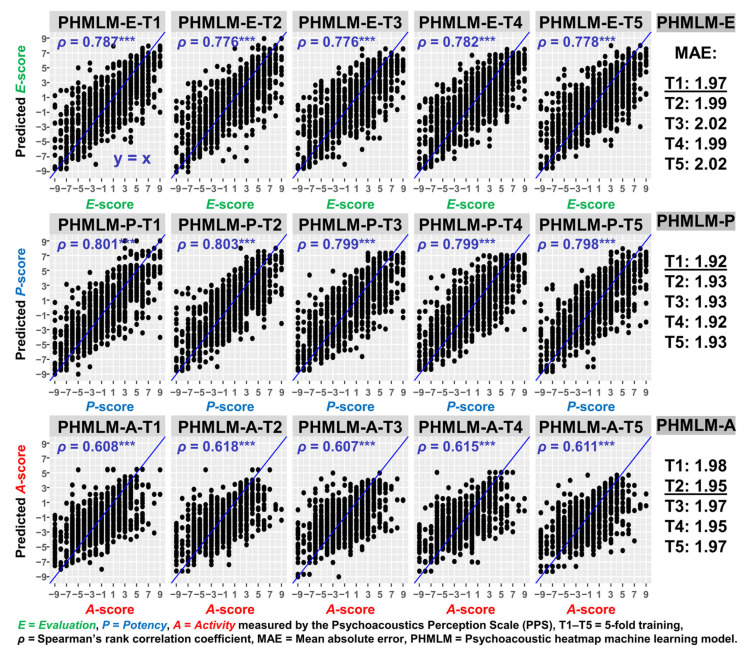
Predictive performance of the psychoacoustic heatmap machine learning models (PHMLM-E, PHMLM-P, and PHMLM-A) for predicting the environmental sound quality in terms of *E*-, *P*-, and *A*-scores in 5-fold training (T1–T5). *** *p*-value < 0.001 in a bivariate correlation test. Models achieving the lowest MAE values for *E*-, *P*-, and *A*-score predictions are underlined.

The excellent internal consistency of the semantic pairs in the *E-*, *P-*, *A-*, and *EPA-*dimensions was found by their Cronbach’s α values of 0.93, 0.90, 0.90, and 0.90, respectively. These high α values supported the reliability of the items in constructing the *E-*, *P-*, *A-*, and *EPA-*scores. The final predictive performance of PHMLM-E, PHMLM-P, and PHMLM-A for predicting the environmental sound quality in terms of *E*-, *P*-, and *A*-scores in 5-fold training (T1–T5) after 4000 epochs is shown in Figure 4.

For the predicted *E*- and *P*-scores from PHMLM-E and PHMLM-P, significant correlations with Spearman’s rank correlation coefficients (*ρ*) consistently around 0.78–0.80 (*p* values < 0.001) and mean absolute errors (MAE) ranging from 1.92 to 2.02 were found between the predicted scores and the labelled scores from the jury listening test. For the predicted *A*-score from PHMLM-A, correlations were also found to be significant with the lower *ρ* values between 0.607 and 0.618 and MAEs from 1.95 to 1.98. The scatterplots for all folds (T1–T4) show predicted scores closely aligning with observed values along the y = x reference line, indicating good model accuracy.

#### 3.2.2. Bivariate Correlation Test Results

The PHMLM-EPA was constructed using PHMLM-E-T1, PHMLM-P-T1, and PHMLM-A-T2 models. These models achieved the lowest MAE for *E*-, *P*-, and *A*-score predictions, respectively, across all 1208 jury listening tests among the five models in 5-fold training (see Figure 4). TRM-EPA was based on the result of a stepwise linear regression of the *EPA*-score using all objective acoustic metrics and psychoacoustic metrics as input independent variables (adjusted *R^2^* = 0.32).

(9) $$TRM-EPA=0.66\times RC-0.11\times \left(N_{5}-N_{95}\right)+0.14\times R_{95}-0.15\times L_{N}-43.3$$

**Figure 5:**
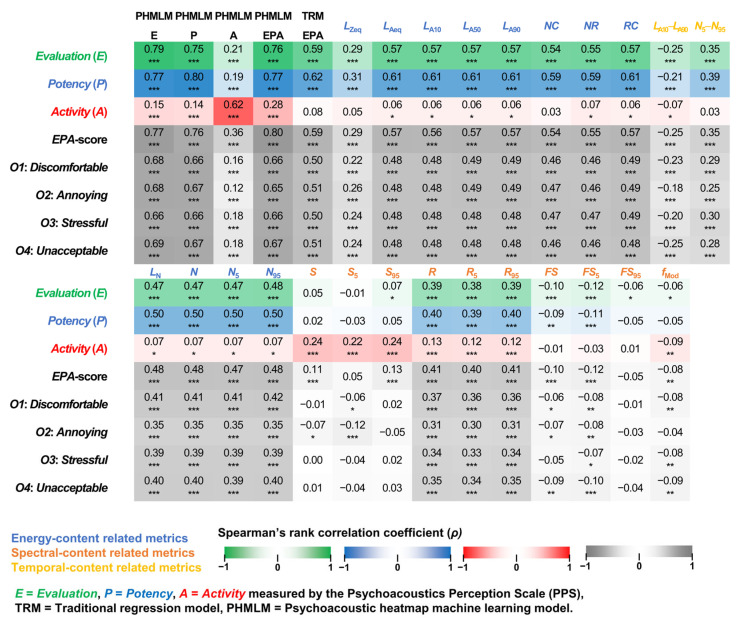
Spearman’s rank correlation coefficient (*ρ*) obtained from the bivariate correlation tests between the individual metrics, model-predicted perceptual scores, and observed perceptual responses to soundtracks (*n* = 1208) recorded in air-conditioned building environments. * *p*-value < 0.05; ** *p*-value < 0.01; *** *p*-value < 0.001 in a bivariate correlation test.

Bivariate correlation tests were conducted between the subjective perceptual responses and the model-predicted perceptual scores (PHMLM-E, PHMLM-P, PHMLM-A, PHMLM-EPA, and TRM-EPA) and individual acoustic and psychoacoustic metrics. Among the individual metrics, significant positive correlations (*p*-values < 0.05, see Figure 5) were found between the energy-content-related metrics (*L*_Zeq_, *L*_Aeq,_ *L*_A10_, *L*_A50,_ *L*_A90_, *NC*, *NR*, *RC*, *L*_N_*, N*, *N*_5_, and *N*_95_) and the subjective *E*-, *P*-, and *EPA*-scores. Moreover, the results indicated significant positive correlations between the values of spectral-content-related metrics (*S*, *S*_5_, *S*_95_, *FS*, *FS*_5_, and *FS*_95_) and the subjective *A*-score.

PHMLM predictions explained a greater proportion of variance in *E*-, *P*-, and especially *A*-scores than TRM-EPA and individual metrics, as shown by higher *ρ* values for *E* (up to 0.79), *P* (up to 0.80), and *A* (up to 0.62) in PHMLM predictions compared to TRM prediction (*E*: 0.59, *P*: 0.62, *A*: 0.28). The higher *ρ* values were also found between PHMLM predictions and the other negative noise impacts (*O1*: *Discomfortable*, *O2*: *Annoying*, *O3*: *Stressful*, and *O4*: *Unacceptable*) than that of TRM prediction and individual metrics.

#### 3.2.3. Predictive Performance of PHMLM-EPA

**Figure 6:**
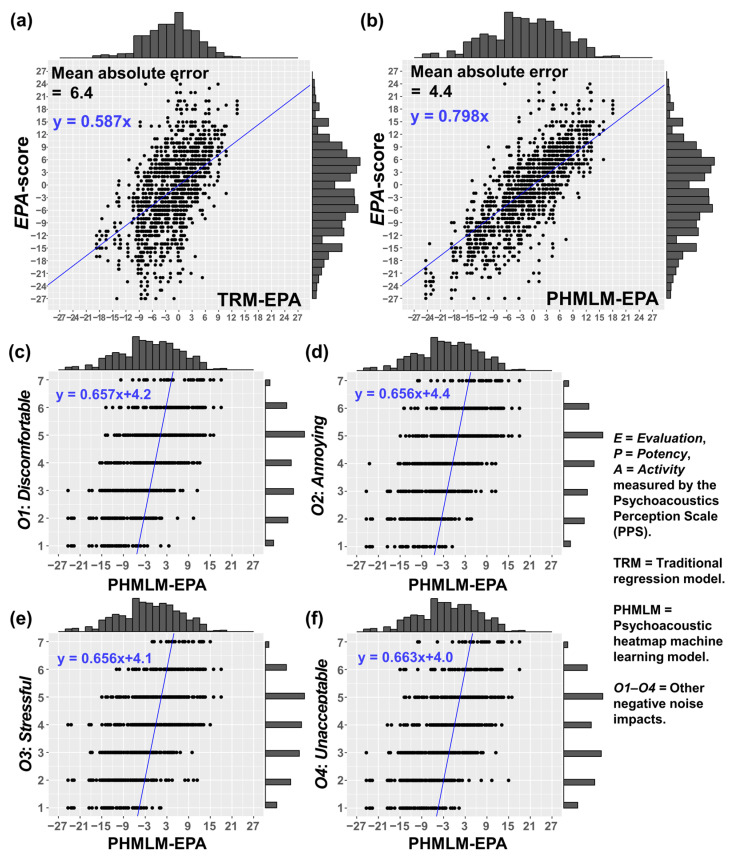
Predictive performance of (**a**) traditional regression model (TRM) on predicting *EPA*-score and (**b**) psychoacoustic heatmap machine learning model (PHMLM) on predicting *EPA*-score and (**c**–**f**) the other negative noise impacts (*O1*: *Discomfortable*, *O2*: *Annoying*, *O3*: *Stressful*, and *O4*: *Unacceptable*).

Predictive performance of PHMLM-EPA (see Figure 6b) on predicting *EPA*-score was found to be better than that of TRM-EPA (see Figure 6a), yielding a lower mean absolute error (4.4 vs. 6.4) and a higher regression slope (0.798 vs. 0.587), with predicted scores more closely aligning with observed values (see Figure 6a,b). In addition, PHMLM-EPA was found to be a significant predictor (*p*-values < 0.05) of all the other negative noise impacts (*O1*: *Discomfortable*, *O2*: *Annoying*, *O3*: *Stressful*, and *O4*: *Unacceptable*), with linear regression slopes of about 0.66 and intercepts near 4.0–4.4 (see Figure 6c–f).

The linear regression results showed that PHMLM-EPA was found to be a significant predictor of the observed *EPA*-score (adjusted *R^2^* = 0.632, F (1, 1207) = 2070, *p-*value < 0.001), *O1*: *Discomfortable* (adjusted *R^2^* = 0.431, F (1, 1207) = 917, *p-*value < 0.001), *O2*: *Annoying* (adjusted *R^2^* = 0.430, F (1, 1207) = 912, *p-*value < 0.001), *O3*: *Stressful* (adjusted *R^2^* = 0.430, F (1, 1207) = 910, *p-*value < 0.001), and *O4*: *Unacceptable* (adjusted *R^2^* = 0.439, F (1, 1207) = 926, *p-*value < 0.001). Table 4 shows that the data-driven PHMLM approach provided more accurate and reliable prediction on the overall sound quality and negative impacts for air-conditioned building environments compared to TRM-EPA (*EPA*-score: *R^2^* = 0.324, F (1, 1207) = 580, *p-*value < 0.001; *O1*: *R^2^* = 0.264, F (1, 1207) = 434, *p-*value < 0.001; *O2*: *R^2^* = 0.278, F (1, 1207) = 466, *p-*value < 0.001; *O3*: *R^2^* = 0.272, F (1, 1207) = 451, *p-*value < 0.001; *O4*: *R^2^* = 0.266, F (1, 1207) = 428, *p-*value < 0.001). Compared to the models relying solely on an individual acoustic or psychoacoustic metric, the improvement in prediction using the predicted *EPA-*score provided by PHMLM-EPA is also shown in Table 4.

## 4. Discussion

### 4.1. Multidimensional Acoustic Characteristics Captured by a Psychoacoustic Heatmap

The results of this study aligned with prior studies showing that human perceptions are multidimensional responses influenced by energy, spectral, and temporal content of acoustic environments [53]. The results of the bivariate correlation tests (*p*-values < 0.05, see Figure 5) revealed that *E*-dimension correlated with all energy-, spectral-, and temporal-content-related metrics, *P*-dimension with energy-content related metrics, and *A*-dimension with spectral-content related metrics, especially *S* and *N*_5_*–N*_95_.

The psychoacoustic heatmap approach introduced in this study representes a significant advancement in acoustic monitoring by transforming time-varying acoustic characteristics into comprehensive visual representations that capture the energy-, spectral-, and temporal-content-related information of sounds. The conversion of psychoacoustic metrics *N*, *S*, *R*, and *FS* into 227 × 227-pixel intensity maps addressed a fundamental limitation of traditional acoustic assessment methods that rely on single-value metrics. As demonstrated by recent research in building acoustics, traditional approaches using averaged sound pressure levels were insufficient to capture the dynamic acoustic characteristics that influence human perception and noise impacts in built environments [53]. The intensity scaling used in this study (25.5 sone for *N*, 2.55 acum for *S*, 0.31875 asper for *R*, and 0.255 vacil for *FS*) provided sufficient resolution to represent the full range of psychoacoustic variations typically encountered in air-conditioned building environments. The temporal resolution of 0.002 s per pixel ensured that rapid fluctuations in HVAC operation, such as compressor cycling and variable fan speeds, were adequately captured in the heatmap representation.

The holistic coverage of energy content (*N*), spectral content (*S*), and temporal content (*R* and *FS*) characteristics in the psychoacoustic heatmaps provided a more holistic representation of the acoustic environment compared to traditional metrics. This multidimensional approach is particularly relevant for HVAC systems, which generate complex acoustic signatures encompassing both steady-state operation and transient events such as startup, shutdown, and mode switching. The significant correlations found between *N*, *S*, *R*, and *FS* and *E*-, *P*-, and *A*-dimensions (*p*-values < 0.05, see Figure 5) confirmed the effectiveness of psychoacoustic heatmaps in capturing perceptually relevant acoustic characteristics.

### 4.2. Integration of Machine Learning and EPA Model

The integration of neural network architectures with the EPA model represented a novel contribution to building acoustics research, providing more interpretable multidimensional predictions of human perceptual responses to the sound quality of air-conditioned building environments than traditional methods. The EPA model’s foundation in three fundamental human perceptual dimensions provided a scientifically validated framework for quantifying sound quality that extends beyond traditional noise level assessment. The excellent internal consistency demonstrated by Cronbach’s α values exceeding 0.90 for all EPA dimensions validated the reliability of the psychometric tool for acoustic monitoring applications. This reliability is crucial for establishing the EPA model as a standardized assessment framework that can be consistently applied across different building types and HVAC configurations. The strong correlations between *EPA*-scores and negative noise impact assessments (*O1*-*O4*) further confirmed the model’s effectiveness in predicting occupant responses to air-conditioned noise, addressing a critical need identified in building environmental quality research.

Transfer learning with pre-trained AlexNet leveraged computer vision for acoustic analysis. The 19-level output layer (−9 to +9) maintained pre-trained feature extraction while enabling task-specific learning for sound quality prediction. The noise features stored in psychoacoustic heatmaps affecting the sound quality perceptions were then learned and updated during each model training iteration.

### 4.3. Predictive Performance of PHMLM

The better predictive performance of PHMLM demonstrated the value of deep learning for capturing non-linear acoustic–perceptual relationships. Correlation coefficients (PHMLM-E: *ρ* = 0.79; PHMLM-P: 0.80; PHMLM-A: 0.62; PHMLM-EPA: 0.80, see Figure 4) exceeded traditional single-metric methods (see Figure 5) and established new benchmarks for sound quality prediction in acoustic sensing. However, PHMLM-A performance (*ρ* = 0.607–0.618) was lower than PHMLM-E and PHMLM-P (0.776–0.803), consistent with prior studies [15,53]. *A*-dimension perception requires temporal and spectral variation; however, HVAC sounds showed lower spectral variability (e.g., IQR/Median of *S* = 0.022/1.16 = 0.19) than energy variability (e.g., IQR/Median of *N* = 4.93/7.26 = 0.68). Future studies should test PHMLM-A on sounds with greater temporal and spectral variation. If PHMLM-A remains satisfactory compared to PHMLM-E and PHMLM-P models, weighting in PHMLM-EPA combination should be adjusted, as equal weights were used in this study.

The 5-fold training methodology employed in this study provided evidence against overfitting while ensuring the generalizability of the trained models. This approach addressed a critical concern in machine learning applications where models may achieve high accuracy on training data but fail to generalize to new situations. The consistent performance across all five folds demonstrates the stability of the PHMLM approach and suggests that the 51,529 pixels of psychoacoustic information contained in each heatmap provide sufficient feature richness to support reliable prediction without memorization of training-specific patterns.

The MAE of 4.4 for *EPA*-score prediction of PHMLM-EPA compared to 6.4 for TRM-EPA represented a 31% improvement in prediction accuracy (see Figure 6), which is practically significant for sound quality prediction in acoustic monitoring. The higher goodness-of-fit demonstrated by PHMLM-EPA for predicting negative noise impacts (adjusted *R^2^* values of 0.43–0.44) compared to TRM-EPA and traditional acoustic metric *L*_Aeq_ demonstrated improvements of 55–95% and 87–95%, respectively.

### 4.4. Implications for Building Design and Engineering Practice

The practical implications of PHMLM for building engineering practice are substantial, particularly in the context of sustainable building design [60] and HVAC system optimization. The ability to predict occupant perceptual responses using only 30 s acoustic recordings provides building engineers with a rapid acoustic monitoring tool that can be deployed during design development, commissioning, and post-occupancy evaluation phases. This capability addresses the time and cost constraints that often prevent comprehensive acoustic monitoring in building projects.

The framework’s potential integration with building information modelling and computational design workflows provides an opportunity for proactive acoustic design rather than reactive noise control. By enabling early-stage prediction of occupant responses to HVAC configurations, PHMLM can inform design decisions that optimize both energy performance and acoustic comfort. This dual optimization is particularly relevant given the increasing emphasis on indoor environmental quality in green building certification systems and occupant well-being standards.

The evidence-based guidelines that PHMLM can provide for air-conditioned noise control align with current trends toward performance-based building codes and standards. Rather than relying solely on prescriptive noise limits based on *L*_Aeq_, the approach enables assessment of actual occupant impact, supporting more flexible and effective regulatory frameworks. The correlation between PHMLM-EPA and the other noise impacts, *O1*: *Discomfortable*, *O2*: *Annoying*, *O3*: *Stressful*, and *O4*: *Unacceptable* (see Figure 6), provided a comprehensive basis for establishing performance thresholds that reflect real occupant concerns.

Moreover, the PHMLM approach represented a significant advancement over current international guidelines for HVAC noise assessment, which primarily rely on single-number ratings such as *NC*, *NR*, and *RC* curves. While these traditional methods provided standardized frameworks for noise control, they failed to account for the temporal variations and spectral complexities that influence human perceptions of air-conditioned sounds. The EPA model’s incorporation of perceptual dimensions provides a more nuanced assessment framework that better reflects the WHO guidelines emphasizing the importance of how people personally experience environmental noise.

### 4.5. Limitations and Future Work

PHMLM was validated using only university HVAC systems, limiting generalizability. Future work should test performance across residential, commercial, and hospital settings with diverse acoustic conditions and sound types (flow-generated, ductwork, office noise). Extended soundtrack durations and additional psychoacoustic metrics should be evaluated for non-HVAC applications. The controlled laboratory setting enabled systematic investigation but limited ecological validity [61]. Real building environments involve contextual [62], visual [63], and social factors that influence sound perception. While controlling these variables follows scientific protocol, results may not directly transfer to complex real-world settings where multiple factors interact simultaneously.

This study’s demographic scope presents constraints on how broadly the findings can be generalized to diverse populations [64]. By recruiting exclusively university students (mean age 22.6 ± 2.1 years, 71.3% male) from a single institution, the research captures responses from a relatively homogeneous cohort that may not represent occupants across age ranges, cultural backgrounds, socioeconomic statuses, or work experience. Previous research has demonstrated that age, cultural heritage, noise exposure history, and geographic origin significantly influence sound perception and noise impact assessment [65], yet these factors remain uncontrolled in the current sample. The PHMLMs thus reflect acoustic comfort preferences of young, tertiary-educated listeners in a specific cultural context, limiting confidence in how effectively the trained models generalize to diverse building occupants globally. To address generalization limitations, systematic external validation should be conducted in field settings with diverse participant demographics and building types. Multi-site replication studies incorporating participants from different age cohorts (children, working adults, elderly), cultural backgrounds, and occupational exposure histories would reveal whether PHMLM’s predictive accuracy holds across heterogeneous populations. Parallel acoustic monitoring in residential HVAC systems, commercial offices, hospitals, and mixed-mode buildings would determine whether model transfer or recalibration is necessary. Furthermore, comparative analysis between PHMLM trained on university data vs. models retrained on diverse populations would quantify potential prediction bias and establish performance thresholds for safe implementation across unfamiliar building contexts.

In this study, only the visual representation of sound using psychoacoustic heatmaps based on four psychoacoustic metrics was considered. Future research could compare the performance of PHMLM with machine learning models that use classical visual representations such as the Mel-spectrogram, psychoacoustic heatmaps with additional metrics (e.g., tonality, prominence ratio, Zwicker loudness patterns), higher temporal resolution heatmaps, or different combinations of acoustic and psychoacoustic metrics as input for noise impact prediction. Moreover, systematic comparisons with existing psychoacoustic machine learning frameworks that rely on time-averaged psychoacoustic metrics (e.g., stationary loudness, sharpness, and roughness [56,57,58]) would quantify PHMLM’s advantages in capturing temporal psychoacoustic evolution for non-stationary indoor noise environments. In addition, future work can be conducted to compare the predictive performance of PHMLM between configurations using psychoacoustic heatmaps and Mel-spectrograms as input.

Although PHMLM demonstrated better performance against the TRM (standardized analysis method [23]) for noise impact prediction, direct comparisons with other commonly applied machine learning baselines such as convolutional neural networks (CNNs), support vector machines (SVMs) with radial basis function (RBF) kernels, Random Forests, and multilayer perceptrons (MLPs) for objective signal classification tasks [41,42,43,44,45] were not conducted in this study. These models exhibit high heterogeneity across studies due to variations in architecture, hyperparameters, and data preprocessing, particularly challenging for psychoacoustic spectrogram inputs. Future work will benchmark PHMLM against CNNs (for spatial feature extraction from spectrograms), SVMs with RBF kernels (for non-linear *EPA*-score boundaries), Random Forests (to assess ensemble performance on stratified folds), and MLPs (as fully connected counterparts to PHMLM’s hybrid architecture). The PHMLM will also be benchmarked against alternative pre-trained deep neural networks such as ResNet, GoogLeNet, Inception Net, and VGGNet architectures to evaluate transfer learning performance across different network depths and complexities for noise impact prediction. The predictive performance of PHMLM will be compared to those models, establishing PHMLM’s contribution within the broader machine learning framework for noise impact prediction.

Several important research directions should be conducted to address these limitations and strengthen the applicability of this work. Field studies conducted in actual building environments would provide much stronger ecological validity by incorporating the full complexity of real-world multisensory experiences and contextual factors that people encounter daily. Expanding participant recruitment to include people across different age ranges, cultural backgrounds, occupational histories, and geographic origins would significantly improve the models’ ability to generalize across diverse populations and reveal how different groups may perceive sounds differently. Conducting replication studies across multiple locations, building types, and cultural contexts would help determine if the PHMLM methodology remains valid and transferable under varying conditions while preserving the core benefits of the psychoacoustic heatmap methodology.

## 5. Conclusions

This study presentes a novel and data-driven methodology (PHMLM) for predicting perceptual sound quality and noise impacts in air-conditioned building environments from objective input of acoustic characteristics. The training of a neural network for machine learning was based on holistic multidimensional sound quality assessments. The bivariate correlation tests between objective acoustic characteristics and subjective perceptual responses (*p*-values < 0.05) showed that the human general judgement of sounds (*E*-dimension) was significant correlated with most of the energy-, spectral-, and temporal-content-related acoustic and psychoacoustic metrics; the sensation of sound energy content (*P*-dimension) was significantly correlated with the energy-content-related metrics; and the sensation of temporal and spectral content (*A*-dimension) was significantly correlated with the temporal- and spectral-content-related metrics, especially *S* and *N*_5_*–N*_95_. The 227 × 227 psychoacoustic heatmaps provided a possible solution to convert both the energy, spectral, and temporal content of the sounds into intensity maps of the time-varying psychoacoustic metrics *N*, *S*, *R*, and *FS* in 51,529 pixels. From the internal consistency test results, Cronbach’s α values > 0.9 (excellent internal consistency) suggested that the PPS was a reliable tool in modelling the fundamental human perceptual dimensions of sound in terms of *E*-, *P*-, and *A*-scores. These results also provided reliable sound quality labelling for the psychoacoustic heatmaps in machine learning.

The best predictive performance of PHMLM-E, PHMLM-P, and PHMLM-A for predicting *E*-, *P*-, and *A*-scores achieved *ρ* = 0.79, 0.80, and 0.62 (*p*-values < 0.05) after 5-fold training. The *ρ* values were higher than that of any individual single-value metrics (*L*_Aeq_: 0.57; *L*_Aeq_: 0.61; *S*: 0.24) in predicting the *E*-, *P*-, and *A*-scores. Although the stepwise linear regression suggested the multidimensional metric selection (energy-content-related: *RC* and *L*_N_; spectral-content-related: *R*_95_; temporal-content-related: *N*_5_*–N*_95_), the selected metric was still based on the single-value metrics and would be different for different sound types and limited in number. In contrast, the 227 × 227 psychoacoustic heatmap can capture the multidimensional acoustic characteristics of 30 s soundtracks and can be a universal objective input in future building acoustics studies. Moreover, the predictive performance of PHMLM-EPA on predicting *EPA*-score was found to be better than that of TRM-EPA with a lower mean absolute error (4.4 vs. 6.4) and a higher regression slope (0.798 vs. 0.587).

For the noise impact predictions of the other negative noise impacts (*O1*: *Discomfortable*, *O2*: *Annoying*, *O3*: *Stressful*, and *O4*: *Unacceptable*), PHMLM-EPA demonstrated a higher goodness-of-fit, as indicated by the adjusted *R^2^* values, than TRM (+55% to +95%) and traditional acoustic metric *L*_Aeq_ (+87% to 95%). The PHMLM delivered more reliable and interpretable multidimensional predictions of human perceptual responses to the sound quality of air-conditioned building environments than traditional acoustic monitoring. Its engineering-based, user-oriented approach offers actionable, evidence-based guidelines to support sustainable building design, operation, and certification by simply using a psychoacoustic heatmap of a 30 s soundtrack as input.

## Figures and Tables

**Table 1:** Descriptive statistics of the acoustic characteristics of the soundtracks (*n* = 1208) recorded from air-conditioned building environments in terms of nine acoustic metrics.

Metric	Unit	Mean	SD	95% CI	25%Tile	Median	75%Tile	IQR
*L*_Zeq_	dB	68.4	5.82	[68.1, 68.8]	64.5	68.6	72.5	8.00
*L*_Aeq_	dBA	48.6	5.91	[48.3, 48.9]	44.1	48.7	53.0	8.88
*L*_A10_	dBA	49.0	5.89	[48.7, 49.3]	44.4	49.1	53.5	9.08
*L*_A50_	dBA	48.5	5.98	[48.1, 48.8]	44.0	48.6	52.9	8.89
*L*_A90_	dBA	48.1	6.02	[47.7, 48.4]	43.5	48.2	52.6	9.08
*L*_A10_ *–L*_A90_	dBA	0.94	0.46	[0.91, 0.96]	0.68	0.78	0.93	0.25
*NC*	NC	45.2	6.04	[44.9, 45.5]	41.0	46.0	50.0	9.00
*NR*	NR	46.0	5.67	[45.7, 46.3]	42.0	46.0	50.0	8.00
*RC*	RC	45.2	5.75	[44.9, 45.6]	41.0	46.0	50.0	9.00

Notes. *n* = number of soundtracks, *L*_Z_ = unweighted sound pressure level, *L*_A_ = A-weighted sound pressure level, *NC* = Noise Criteria, *NR* = Noise Rating, *RC* = Room Criteria, _eq_ = time-equivalent, _5/10/50/90/95_ *=* percentiles of 5/10/50/90/95%, SD = standard deviation, CI = confidence interval for mean, %tile = percentile, IQR = interquartile range.

**Table 2:** Descriptive statistics of the acoustic characteristics of the soundtracks (*n* = 1208) recorded from air-conditioned building environments in terms of fifteen psychoacoustic metrics.

Metric	Unit	Mean	SD	95% CI	25%Tile	Median	75%Tile	IQR
*L*_N_	phon	68.7	7.93	[68.2, 69.1]	63.4	68.6	73.2	9.81
*N*	sone	8.50	5.20	[8.21, 8.8]	5.07	7.26	10.00	4.93
*N*_5_	sone	8.81	5.34	[8.51, 9.12]	5.45	7.62	10.44	4.99
*N*_95_	sone	7.83	4.87	[7.55, 8.1]	4.67	6.70	9.32	4.65
*S*	acum	1.13	0.19	[1.12, 1.14]	1.02	1.16	1.25	0.22
*S*_5_	acum	1.24	0.21	[1.23, 1.25]	1.13	1.25	1.35	0.22
*S*_95_	acum	1.05	0.19	[1.04, 1.06]	0.93	1.08	1.17	0.24
*R*	asper	0.08	0.02	[0.08, 0.08]	0.07	0.08	0.10	0.03
*R*_5_	asper	0.13	0.04	[0.12, 0.13]	0.10	0.12	0.15	0.04
*R*_95_	asper	0.05	0.01	[0.05, 0.05]	0.04	0.05	0.06	0.02
*FS*	vacil	0.03	0.02	[0.02, 0.03]	0.01	0.02	0.04	0.03
*FS*_5_	vacil	0.03	0.03	[0.03, 0.04]	0.01	0.02	0.05	0.04
*FS*_95_	vacil	0.02	0.02	[0.02, 0.02]	0.004	0.01	0.03	0.02
*f*_Mod_	Hertz	131	35	[129, 133]	111	131	150	39
*N*_5_–*N*_95_	sone	0.99	0.55	[0.96, 1.02]	0.64	0.86	1.12	0.48

Notes. *n* = number of soundtracks, _5/10/50/90/95_ *=* percentiles of 5/10/50/90/95%, SD = standard deviation, CI = confidence interval for mean, %tile = percentile, IQR = interquartile range, *N* = total loudness, *S* = sharpness, *R* = roughness, *FS* = fluctuation strength, *f*_Mod_ = dominant modulation frequency determined in the calculation of *R*.

**Table 3:** Details of all hyperparameters in training options (‘trainingOptions’, MATLAB R2024a) for psychoacoustic heatmap machine learning models (PHMLM-E, PHMLM-P, and PHMLM-A).

Parameter	Value/Type	Description/Justification
Solver	sgdm	Stochastic Gradient Descent with Momentum
Momentum	0.95	Standard for stable convergence
InitialLearnRate	0.001	Optimal for transfer learning
LearnRateSchedule	‘piecewise’	Step decay for long-term convergence
LearnRateDropPeriod	400	Drop every 400 epochs (10× MiniBatchSize)
LearnRateDropFactor	0.6	Conservative decay (60% reduction)
MiniBatchSize	80	Balances memory/gradient stability
MaxEpochs	4000	Required by piecewise schedule (10 drops)
Shuffle	‘every-epoch’	Prevents overfitting standard practice
ValidationFrequency	12	Every 12 iterations (efficient monitoring)
ValidationPatience	Inf	No early stopping; relies on learning rate schedule
Verbose	false	Clean output

**Table 4:** Percentage improvement of the adjusted *R^2^* of the psychoacoustic heatmap machine learning model (PHMLM-EPA) as a predictor for *EPA*-score and other negative noise impacts compared to the traditional regression model (TRM)and individual acoustic and psychoacoustic metrics.

Dependent Variable	PHMLM-EPA (Adjusted *R*^2^)	TRM-EPA	*L*_Aeq_	*L*_A10_	*L*_A50_	*L*_A90_	*NC*	*NR*	*RC*	*L*_N_	*N*	*N*_5_	*N*_95_	*R*	*R*_5_	*R*_95_
*EPA*-score	0.63	95%	95%	102%	95%	95%	117%	109%	95%	174%	174%	186%	174%	276%	295%	276%
*O1*: *Discomfortable*	0.43	63%	87%	87%	80%	80%	104%	104%	80%	156%	156%	156%	144%	215%	233%	233%
*O2*: *Annoying*	0.43	55%	87%	87%	79%	79%	95%	103%	79%	251%	251%	251%	251%	347%	378%	347%
*O3*: *Stressful*	0.43	58%	87%	87%	87%	87%	95%	95%	79%	183%	183%	183%	183%	272%	295%	272%
*O4*: *Unacceptable*	0.44	65%	91%	91%	91%	91%	107%	107%	91%	174%	174%	189%	174%	258%	280%	258%

Notes. *L*_A_ = A-weighted sound pressure level, *NC* = Noise Criteria, *NR* = Noise Rating, *RC* = Room Criteria, *N* = total loudness, *R* = roughness, _eq_ = time-equivalent, _5/10/50/90/95_ *=* percentiles of 5/10/50/90/95%, *O1*–*O4* = other negative noise impacts.

## Data Availability

The raw data supporting the conclusions of this article will be made available by the authors on request. MATLAB (.mat) files of the trained neural networks of psychoacoustic heatmap machine learning model (PHMLM) for predicting perceptual sound quality can be made available upon reasonable request.

## Abbreviations

The following abbreviations are used in this manuscript:

PHMLM	Psychoacoustic Heatmap Machine Learning Models
E	Evaluation
P	Potency
A	Activity
EPA	Evaluation, Potency, and Activity
PPS	Psychoacoustics Perception Scale
TRM	Traditional Regression Models
WHO	World Health Organization
NC	Noise Criteria
NR	Noise Rating
RC	Room Criteria
ANN	Artificial Neural Network
FC	Fully Connected
MP	Max Pooling
conv	Convolutional
CI	Confidence Interval
SD	Standard Deviation
IQR	Interquartile Range
MAE	Mean Absolute Errors
CNN	Convolutional Neural Network
SGDM	Stochastic Gradient Descent with Momentum
SVM	Support Vector Machines
RBF	Radial Basis Function
MLPs	Multilayer Perceptrons

## Appendix A

### Appendix A.1. Equations of the Acoustic Metrics

(A1) $$L_{Zeq}=10\;{\log_{10}\left(\frac{\sqrt{\frac{1}{T}\int_{0}^{T}p^{2}\left(t\right)dt\;}}{p_{0}}\right)}^{2}(dB),$$

where *L*_zeq_ is zero-weighted equivalent sound pressure level, *T* is an elapse time, *p*(t) is the sound pressure at time *t* in pascals (Pa), and *p*_0_ is the reference sound pressure (20 μPa).

(A2) $$L_{Aeq}=10\;{\log_{10}\left(\frac{\sqrt{\frac{1}{T}\int_{0}^{T}p_{A}^{2}\left(t\right)dt\;}}{p_{0}}\right)}^{2}=10{log}_{10}\left(\frac{1}{M}\sum_{m=1}^{M}10^{\frac{L_{Am}}{10}}\;\;\right)\;\left(dBA\right),$$

where *L*_Aeq_ is A-weighted equivalent sound pressure level, *L*_Am_ is the mth instantaneous A-weighted sound pressure level of the detector response time (Fast response time = 125 ms), and *M* = elapse time/response time.

*L*_AP%_ is the instantaneous A-weighted sound pressure level of the detector response time (Fast response time = 125 ms) that exceeds P% of the elapse time.

### Appendix A.2. Equations of the Psychoacoustic Metrics

(A3) $$N^{\prime }\left(t\right)=0.08\;{\left(\frac{E_{TQ}}{E_{0}}\right)}^{0.23}\left[{\left(0.5+0.5\frac{E\left(t\right)}{E_{TQ}}\right)}^{0.23}-1\right]\left(\frac{sone}{Bark}\right),$$

where *N’(t)* is the time-varying specific loudness in sone per Bark, *E(t)* is time-varying excitation of the sound, *E*_TQ_ is excitation of threshold in quiet, and *E*_0_ is the reference excitation. Loudness of a 1 kHz pure tone sound at 40 dB is defined to be 1 sone and 40 phon.

(A4) $$N(t)=\int_{0}^{24Bark}N^{\prime }(t)dz\;\;(sone),$$

where *N(t)* is the time-varying total loudness.

(A5) $$S\left(t\right)=0.11\frac{\int_{0}^{24Bark}N^{\prime }\left(t\right)g\left(z\right)zdz}{\int_{0}^{24Bark}N^{\prime }dz}\left(acum\right),$$

(A6) $$g\left(z\right):=\left\{\begin{array}{c} 1,\;\;z\leq 14\\ 0.00012Z^{4}-0.0056Z^{3}+0.1Z^{2}-0.81z+3.51,\;\;z>14 \end{array}\right.$$

where *S(t)* is the time-varying sharpness; *g(*z*)* is critical-band rate. The sharpness of a 1 kHz pure tone sound at 60 dB is defined to be 1 acum.

(A7) $$R=0.0003f_{Mod}\int_{0}^{24\;Bark}∆L\left(z\right)dz\left(asper\right),$$

(A8) $$FS=0.008\frac{\int_{0}^{24\;Bark}∆L\left(z\right)dz}{\left(\frac{f_{Mod}}{4}+\frac{4}{f_{Mod}}\right)}\left(vacil\right),$$

where *f*_Mod_ is the dominant modulation frequency for the effects of frequency resolution (Hz), and Δ*L* is the temporal depth for masking the effect of temporal resolution (dB). A 1 kHz pure tone with at 60 dB 100% amplitude modulation of 70 Hz and of 4 Hz is equal to 1 asper and 1 vacil, respectively.

For the calculations of *N*, the time-averaged excitation of sound is utilized.

(A9) $$L_{N}=\left\{\begin{array}{c} 40\;{\left(\frac{N}{sone}+0.005\right)}^{0.35},\;\;N<1\;sone\\ 40+10{log}_{2}(\frac{N}{sone}),\;\;N\geq 1\;sone \end{array}\right.(phon)$$

where *L*_N_ is the loudness level converted from the time-averaged *N* in phon.

*N*_P%_ and *S*_P%_ are the values of the instantaneous total loudness and sharpness that exceed P% of the elapse time, respectively.

### Appendix A.3. Equations of Statistical Analysis

Spearman’s rank correlation coefficient (*ρ*) is a statistical measure used to determine the strength and direction of the linear relationship between two variables, ranging from –1 to 1.

(A10) $$\rho =1-\frac{6\sum d_{i}^{2}}{n(n^{2}-1)},$$

where *n* is total sample size; *d*_i_ is difference between the two ranks of each sample.

Adjusted *R^2^* values (coefficient of determination).

(A11) $$y=b_{1}x_{1}+b_{2}x_{2}+b_{3}x_{3}+\cdots b_{n}x_{n}+C$$

(A12) $$Adjusted\;R^{2}=1-\left(1-\frac{\sum_{i=1}^{n}{\left(\hat{y_{i}}-\bar{y}\right)}^{2}\;}{\sum_{i=1}^{n}{\left(y_{i}-\bar{y}\right)}^{2}\;}\right)\times \frac{\left(n-1\right)}{\left(n-p-1\right)},$$

where y is a dependent variable, *b*_1_-*b*_1_ are the regression coefficients for the independent variables (*x_1_*–*x_n_*) as predictors, *C* is the y-intercept, *n* is total sample size, *p* is number of predictors, and $\hat{y_{i}}$ and $\bar{y}$ are the prediction and mean of y.
